# Split-Hand Malformation in a 4-Year-Old Child

**DOI:** 10.1155/2017/6073619

**Published:** 2017-08-03

**Authors:** Girish Gulab Meshram, Kanwaljeet Singh Hura, Neeraj Kaur

**Affiliations:** ^1^Department of Pharmacology, Postgraduate Institute of Medical Education and Research and Dr. Ram Manohar Lohia Hospital, New Delhi, India; ^2^Department of Pediatrics, Richmond University Medical Centre, Staten Island, NY, USA; ^3^Department of Radiology, University of Texas Health Science Centre, San Antonio, TX, USA

## Abstract

Split-hand deformity is one of the milder manifestations of a congenital disorder called split-hand/split-foot malformation. We present a case of a 4-year-old child with split-hand malformation in his left hand since birth. A median cleft was present in the affected hand with absence of the 3rd and 4th digits, giving rise to a characteristic lobster-claw appearance. Functionality of the affected hand was modestly impaired. As none of the close family members of the patient had similar limb malformations, the deformity was postulated to arise most likely from a de novo mutation. The patient was discharged after the parents were provided with genetic counseling.

## 1. Introduction

Split-hand malformation (SHM) is a congenital defect resulting from a chromosomal abnormality in the 7q21q22 region leading to abnormal limb morphogenesis [1]. The presentation of SHM could vary from absence of a single finger to the presence of only one finger (monodactyly) [2]. SHM can occur in isolation or as a part of various complex syndromes [3]. Isolated SHM is usually inherited by the autosomal dominant mode [4]. We report a rare sporadic case of isolated SHM most likely caused by de novo mutation and discuss its etiology, pathogenesis, antenatal diagnosis, genetic counseling, and management.

## 2. Case Presentation

**Figure 1:**
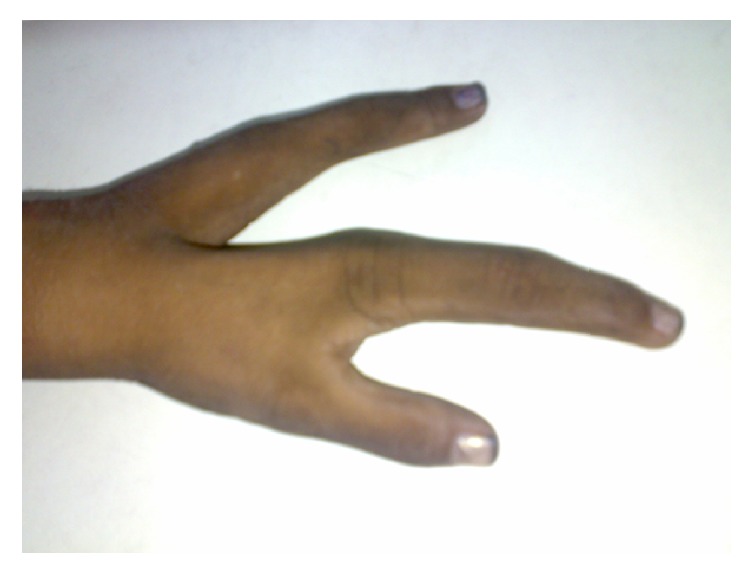
Split-hand malformation with absence of 3rd and 4th digits causing a lobster-claw appearance.

A 4-year-old child presented to the pediatric department with a deformed left hand since birth. The parents of the child had a nonconsanguineous marriage. Our patient was second in birth order. There was no history of similar malformations in the parents, sibling, or close relatives. General and systemic examination of the child appeared unremarkable. On local examination, a median cleft was present on the affected hand. The left hand showed aplasia of the 3rd and 4th digits, giving a characteristic lobster-claw appearance (Figure 1). The X-ray showed normal metacarpals but absence of phalanges of the 3rd and 4th fingers. Language development was appropriate for his age. Audiometric tests ruled out sensorineural hearing loss. A diagnosis of SHM was reached clinically. The routine blood and urine investigations did not show any abnormalities. Karyotyping revealed normal male 46, XY karyotype. Further molecular genetic studies could not be carried out due to the economic constraints of the patient's family. The child was discharged following genetic counseling to the parents.

## 3. Discussion

SHM is one of the milder manifestations of a genetic disorder called split-hand/split-foot malformation (SHFM) [3]. SHFM, depending on varying levels of its manifestations, causes aplasia/hypoplasia of some of the fingers/toes, syndactyly, and presence of median clefts in the affected hands/feet [1–3]. These limb deformities may occur in isolation, as in our case, or in combination with several complex syndromes. SHM, if syndromic, occurs in conjunction with ectodermal dysplasia, cleft lip/palate, mental retardation, and sensorineural deafness [2–4].

SHFM accounts for 8–17% of all limb malformations. The incidence of isolated SHFM is estimated to be 1 per 18000 births, of which 80% have only one affected limb with upper limb predominance [5]. Isolated SHM is caused by mutations at 7q21.3-q22.1, dysregulating the DLX5 and DLX6 genes [3, 4]. DLX6 and DLX5 code transcription factors are largely restricted to the apical ectodermal ridge (AER), a specialized region in the ectoderm required for normal limb skeletal development and morphogenesis [6]. An abnormality in the functioning of the AER leads to abnormalities in the differentiation of the central rays which form the 2nd, 3rd, and 4th digits leading to limb anomalies [5, 6].

SHM is inherited predominantly by the autosomal dominant mode with reduced penetrance, although X-linked and autosomal recessive forms have also been reported [3]. Our case is most likely sporadic in nature as none of the parents, sibling, or close relatives of the patient had similar limb malformations. Also, isolated SHM involving only one upper limb is usually associated with sporadic cases. However, molecular genetic studies as Fluorescence In Situ Hybridization, array-Comparative Genomic Hybridization, and next generation mate-pair sequencing are essential to characterize and truncate the loci of the chromosomal aberrations. These confirmatory genetic studies could not be conducted in our patient. A previous study utilizing the abovementioned molecular genetic techniques stratified SHSF in three subregions around the DLX5/DLX6 location on chromosome 7q21.3: isolated SHSF, SHSF and hearing loss, and SHSF, hearing loss, and craniofacial anomalies [7].

Management of cases of SHFM is aimed at improving functionality and aesthetics of the affected limbs through prosthetics and reconstructive surgeries [8]. Isolated cases of SHM may not require surgical interventions as most patients adapt well and have only a modest functional impairment. Three-dimensional ultrasonography detects SHM as early as the 13th week of gestation [5]. Antenatal genetic diagnostic tests for screening candidate genes have also been suggested for high-risk families. However, commercial availability of genetic testing is limited [3].

In our case, the future siblings of the patient have a low risk of inheriting the disease. However, due to the variability in clinical/genetic expressivity of the disorder, the parents of the patient were advised to follow increased vigilance while planning their next pregnancy. The offspring of the patient have a 30–50% risk of suffering from SHFM as it is predominantly inherited by the autosomal dominant mode and has a higher risk of affecting boys due to skewed transmission with higher penetrance in males [3, 7, 9]. Hence, during the genetic counseling session, the patient's parents were informed in detail of the nature of the disease and the various modalities available for its early detection, prevention, and management.
